# Role of Neurovascular Compression of Oculomotor Nerve in Ophthalmoplegic Migraine

**DOI:** 10.7759/cureus.22919

**Published:** 2022-03-07

**Authors:** Linda T Bui, Gayatra Mainali, Sunil Naik, Kevin Cockroft, Krishnamoorthy Thamburaj

**Affiliations:** 1 Radiology, Penn State College of Medicine, Hershey, USA; 2 Pediatric Neurology, Penn State Health Milton S. Hershey Medical Center, Hershey, USA; 3 Neurosurgery, Penn State Health Milton S. Hershey Medical Center, Hershey, USA; 4 Neuroradiology, Penn State Health Milton S. Hershey Medical Center, Hershey, USA

**Keywords:** ophthalmoplegic migraine, recurrent painful ophthalmoplegic neuropathy, third nerve palsy, oculomotor nerve palsy, migraine

## Abstract

Ophthalmoplegic migraine is considered to occur more commonly in children than in adults. It commonly affects the oculomotor nerve among the cranial nerves. Demyelination of the nerve is proposed as the main mechanism for the etiology of ophthalmoplegic migraine, though it is not fully understood. Neurovascular compression as a cause of ophthalmoplegic migraine has not been well demonstrated in children. In this report, we present a case of a 13-year-old male with recurrent episodes of left ophthalmoplegic migraine. Oculomotor nerve enhancement with swelling was evident on MRI at the exit zone. Magnetic resonance angiography (MRA) revealed a sharp loop of the left posterior cerebral artery compressing the nerve. The case highlighted the unusual etiology of neurovascular compression resulting in ophthalmoplegic migraine in a pediatric patient. A supplemental case of ophthalmoplegic migraine in a seven-year-old male is also shown to highlight the role of neurovascular compression and the importance of using MR angiography to evaluate cases presenting clinically with ophthalmoplegic migraine.

## Introduction

Two or more recurrent migraine-like headaches that involve paralysis of either one or multiple ocular cranial nerves (CN) that occur with either the immediate or delayed onset of pain is defined as an ophthalmoplegic migraine (OM) by the International Headache Society [1]. OM is also referred to more recently as recurrent painful ophthalmoplegic neuropathy [2]. The oculomotor nerve is the most commonly involved nerve in OM, and it often demonstrates enhancement on contrast-enhanced MRI studies [1-4]. Though the exact cause of OM is not well understood, it is thought to occur from recurrent demyelination of the third nerve. The cause of the nerve demyelination is hypothesized to be due to an underlying inflammatory process [1- 2,5]. Some investigators describe a role of neurovascular compression in OM [5-7]. The role of neurovascular compression in painful neuralgias involving the trigeminal nerve and hemifacial spasm involving the facial nerve is well known [8]. We describe two cases of OM with characteristic imaging features resulting from vascular compression at the exit zone of the oculomotor nerve.

## Case presentation

Case 1

**Figure 1:**
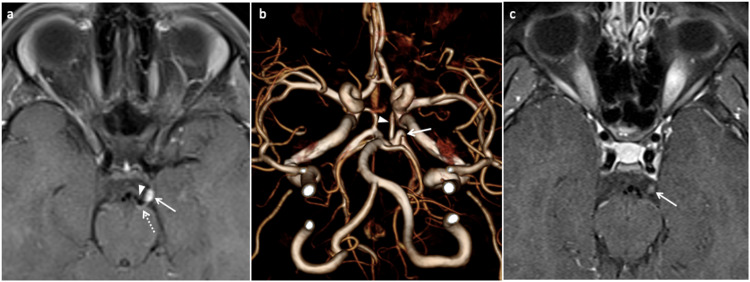
Ophthalmoplegic migraine in a 13-year-old male presenting two weeks after the onset of left eye ptosis. (a) Axial post-contrast T1 TSE fat-saturated image demonstrates enhancement of the left oculomotor nerve at the exit zone near the left cerebral peduncle (arrow). The flow void of the left posterior cerebral artery (triangle) is seen with compression of the nerve against the cerebral peduncle (dotted arrow). (b) Three-dimensional volume rendering of TOF MRA reveals the sharp loop (arrow) in the proximal P2 segment of the left posterior cerebral artery. The left posterior communicating artery (triangle) is joining the left posterior cerebral artery proximal to the loop. (c) Axial T1 TSE fat-saturated image demonstrates a significant decrease in enhancement and swelling of the left oculomotor nerve after recovery (arrow). TSE, turbo spin echo; TOF MRA, time-of-flight magnetic resonance angiograph

A 13-year-old male presented to the emergency department with a two-week history of pain behind his left eye, left pupillary dilatation with sluggish reaction to light, and complete ptosis. His past history was relevant for 10 episodes of left eye ptosis, exotropia, diplopia, and pupillary dilatation of varying severity since the age of three years. He also had prior lab workup for possible underlying autoimmune pathology, which included myasthenia gravis, for which he was negative. The patient had no obvious preceding triggers or illnesses. Each episode resolved on its own or after a short course of oral prednisolone in less than a week. In addition, he also had intermittent headache suggestive of migraine without aura. He had several brain and orbit MRI studies along with a head magnetic resonance angiography (MRA). Imaging revealed an enhancing left third nerve at the exit zone with compression of the nerve by anomalous course of the left posterior cerebral artery (PCA) (Figure 1). Improvement in third nerve enhancement was noted in follow-up MR studies performed after resolution of third nerve palsy. The patient was prescribed topiramate to help treat his migraine-type headaches that occurred without third nerve involvement.

Case 2

**Figure 2:**
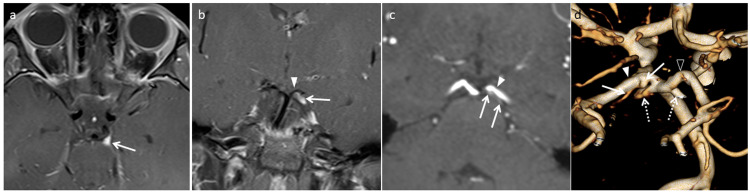
Ophthalmoplegic migraine in a seven-year-old male that recovered completely seven days later. (a) Axial TSE post-contrast fat-saturated image demonstrates swelling and enhancement of the left oculomotor nerve at the exit zone (arrow). (b) Coronal post-contrast T1 TSE fat-saturated image demonstrates enhancing left oculomotor nerve (arrow) between the posterior cerebral artery (triangle) and the superior cerebellar artery. (c) Oblique coronal multi-planar reconstruction image of TOF MRA of the head reveals the anomalous branch with infundibulum at its origin (arrows) from the P1 segment of the left posterior cerebral artery (triangle). The branch is coursing parallel to the left posterior cerebral artery along its posterior margin. (d) Volume rendered reconstruction of TOF MRA demonstrates left posterior cerebral artery (solid triangle), right posterior cerebral artery (triangle) and anomalous branch with infundibulum at its origin from the posterior surface of left posterior cerebral artery (solid arrows). The left posterior cerebral artery is visualized on the right side in the image as it is viewed from the posterior aspect. The superior cerebellar arteries (dotted arrows) have been cut to improve the visualization of anomalous branch from the left posterior cerebral artery. TSE, turbo spin echo; TOF MRA, time-of-flight magnetic resonance angiography

A seven-year-old male with a past medical history of mild autism spectrum disorder presented to the emergency department with a two-day history of acute onset of left eye pain, double vision, and ptosis preceded by a one-week history of viral gastroenteritis. The patient reported photophobia and pain with left eye movement. Pupils were equal and reactive to light. There was complete ptosis and limited extra-ocular movements of the left eye, which was turned laterally and inferiorly, suggesting third nerve palsy. The respiratory viral panel was positive for rhinovirus/enterovirus. An MRI and MRA study of the brain demonstrated an enhanced left third nerve in the cisternal segment, with probable fenestration or anomalous vascular branch of the left PCA near the enhancing nerve (Figure 2). He responded to a course of oral prednisolone with remission of symptoms after a week of steroid.

## Discussion

OM is an uncommon entity, mostly observed in children, with an average age of onset of eight years [1,2]. In OM, third nerve abnormalities on MRI studies are more often described than abnormalities in fourth and sixth CNs [1,4]. In a literature review of 52 cases of OM, MRI enhancement of the third nerve was found in 75% of cases with swelling of the nerve in 76% of cases [1]. Mark et al. reported transient enhancement of the cisternal segment of the third nerve in six cases identified as OM. In five cases, the enhancement was focal and associated with swelling of the nerve near the exit from the midbrain in the interpeduncular cistern. Whole cisternal segment swelling and enhancement of the third nerve was observed in one case. In all cases, the enhancement resolved in seven to nine weeks following a three-week treatment of steroids [3]. In a review on MRI findings in OM, Bharucha et al. reported documentation of third nerve enhancement in 44 of 52 cases from the literature. Furthermore, in their own case of a 16-year-old patient, third nerve enhancement was evident only during the eighth episode of OM. Subsequently, resolution of enhancement was observed three months later [4].

It is important to recognize the characteristic features of third nerve enhancement on MRI in OM to avoid mistaking it for other conditions [1,3,4]. Enhancement of the third nerve may be observed in neoplastic and non-neoplastic conditions [3]. Lymphoma, leukemia, and carcinomatous meningitis are malignant conditions that may cause third nerve enhancement. Enhancement of the third nerve can also be seen in infectious conditions such as Lyme’s disease, syphilis, coccidioidomycosis, and HIV neuritis. Sarcoidosis and Fisher’s syndrome are noninfectious inflammatory causes of third nerve enhancement [1,3,4]. Schwannoma is a well-known benign nerve sheath tumor that may occasionally arise from the third nerve, although it commonly presents as a mass. Resolution of enhancement may be helpful to rule out neoplastic conditions.

Though various mechanisms have been proposed, the pathogenesis behind OM is not well understood [1]. Some consider recurrent demyelination as a potential etiology for OM [1,2,4]. Mark et al. proposed that viral infection or idiopathic inflammatory neuropathy may have caused OM in their series [3], while Liu et al. concluded that an underlying recurrent inflammatory process was the etiology of OM in a review of five cases [2]. A few investigators have reported a role of neurovascular compression of the third nerve in adult-onset OM, although the offered imaging evidence was not convincing in some of the cases [5-7]. Vieira et al. described a case of OM from oculomotor nerve compression by arterial infundibular dilation. However, the infundibulum in this case was located near the internal carotid artery well away from the exit zone of the third nerve [5]. Tocco et al. reported a case of adult-onset OM with cisternal enhancement of the third nerve in association with ipsilateral fetal type PCA. The authors did not provide clear evidence of neural compression on imaging, although they raised the possibility of a role of anomalous arterial branch in the development of OM [6]. OM has also been described in association with compression of the sixth nerve. Linn et al. described a case involving an adult female with recurrent OM with sixth nerve palsy from compression and distortion of the sixth nerve by the basilar artery and the anterior inferior cerebellar artery. The most severe compression of the nerve was observed at its exit point from the brainstem [7].

In our case, the clinical presentation, and MRI and MRA study findings favor the occurrence of OM from neurovascular compression. An illustration of a second case (Figure 2) further lends support to the role of neurovascular compression in the development of OM in children. Recurrent episodes are well known to occur in trigeminal neuralgia, and its relationship to neurovascular compression is well established at the root entry zone of nerve [3,4]. The recurrent episodes in our case, with a spatial relationship of nerve enhancement to the exit zone and compression by PCA, supports the role of neurovascular compression in the development of OM [3,4]. To the best of our knowledge, we came across one report describing neurovascular compression as a cause of OM in children. However, the authors describe infundibulum of the posterior communicating artery at its origin as a cause of OM, which is located well away from the exit zone of the third nerve [5]. The distinct compression and distortion of the third nerve by PCA near the exit shown is well represented in our case (Figure 1) [5-7]. Our cases highlight the importance of searching for neurovascular compression of the third nerve near the exit zone in pediatric-onset OM. This can be accomplished with high-resolution MRA.

## Conclusions

In conclusion, an etiologic role of neurovascular compression needs to be considered in the development of OM in children. High-resolution contrast-enhanced MRI along with MRA can help evaluate the spatial relationship of oculomotor nerve enhancement to the exit zone and its relationship to a potential vascular compression.
